# Autoantigenic peptide landscape of rheumatoid arthritis-associated HLA class II

**DOI:** 10.1016/j.gendis.2024.101469

**Published:** 2024-11-26

**Authors:** Irina A. Ishina, Anton P. Zhiyanov, Inna N. Kurbatskaia, Azad E. Mamedov, Stepan A. Nersisyan, Rustam H. Ziganshin, Igor E. Eliseev, Yunna S. Petrusenko, Anastasia V. Nikonova, Elizaveta S. Zhbanova, Maria A. Salnikova, Leyla A. Ovchinnikova, Ilgar Z. Mamedov, Alexey N. Davydov, Kamila S. Nurbaeva, Tatiana A. Lisitsyna, Tatiana M. Reshetnyak, Alexander M. Lila, Evgeniy L. Nasonov, Yakov A. Lomakin, Alexey A. Belogurov, Hongkai Zhang, Alexander G. Tonevitskiy, Yury P. Rubtsov, Alexander G. Gabibov, Maria Y. Zakharova

**Affiliations:** a Shemyakin-Ovchinnikov Institute of Bioorganic Chemistry, Russian Academy of Sciences, Moscow 117997, Russia; b Faculty of Biology and Biotechnology, Higher School of Economics, University, Moscow 117418, Russia; c Biotech Campus Ltd, Moscow 117437, Russia; d Institute of Translational Medicine, Pirogov Russian National Research Medical University, Moscow 117513, Russia; e Central European Institute of Technology, Brno 60177, Czech Republic; f MiLaboratories Inc, San Francisco, CA 94114, United States; g V.A. Nasonova Research Institute of Rheumatology, Moscow 115522, Russia; h Department of Biological Chemistry, Evdokimov Moscow State University of Medicine and Dentistry, Moscow 127473, Russia; i State Key Laboratory of Medicinal Chemical Biology, College of Life Sciences and Frontiers Science Center for Cell Responses, Nankai University, Tianjin 300071, China; j Department of Chemistry, Lomonosov Moscow State University, Moscow 119991, Russia

Rheumatoid arthritis (RA) is an autoimmune disorder characterized by synovial joint damage and progressive loss of mobility. The human leukocyte antigen (HLA) class II alleles HLA-DRB1∗01:01 and HLA-DRB1∗04:01 are strongly linked to RA susceptibility. Several autoantigenic peptides were reported to bind to RA-associated HLA-II and trigger autoreactive CD4^+^ T cell response. Here, we propose a dual combinatorial approach to identify novel autoantigenic peptides presented by HLA-II. We generated a phage library containing fragments of human autoantigens to screen for peptide ligands binding RA-associated HLA-II. Concurrently, the HLA-II immunopeptidome of peripheral blood mononuclear cells from RA patients was analyzed using liquid chromatography with tandem mass spectrometry (LC-MS/MS). This approach led to the identification of a panel of RA-associated HLA-II peptide ligands, confirmed via *in vitro* binding assay. Identified autoantigens include fragments of annexin A11, endoplasmic reticulum chaperone BiP, calreticulin, and vimentin. Finally, we demonstrated that the annexin A11 fragment, in the complex with HLA-DRB1∗01:01, can activate CD4^+^ T cells from RA patients.

Recombinant HLA-DRB1∗01:01 and HLA-DRB1∗04:01 proteins were used to enrich potential ligands from the phage library. For the library construction, the AAgAtlas database (http://biokb.ncpsb.org/aagatlas) was used as a source of human proteins with documented humoral responses in several autoimmune diseases, including RA, diabetes mellitus, multiple sclerosis, and lupus erythematosus (Fig. 1A). Of note, 104 RA-associated proteins were included in the library. The purpose of utilizing this library to search for HLA-II ligands was to identify novel autoimmune epitopes recognized by CD4^+^ T cells, specifically among B cell epitopes. The phage library contained 11973 44-mer peptides with 14 aa overlaps (Table S1), including 2197 peptides associated with RA (Fig. 1B). Screening of the phage library for binding of HLA-DRB1∗01:01 or HLA-DRB1∗04:01 included two rounds of selection in the presence of the recombinant chaperone HLA-DM, while negative control selection was carried out under the same conditions, but in the absence of HLA-II. The resulting pools of peptides were characterized at each selection step by next-generation sequencing.

**Figure 1:**
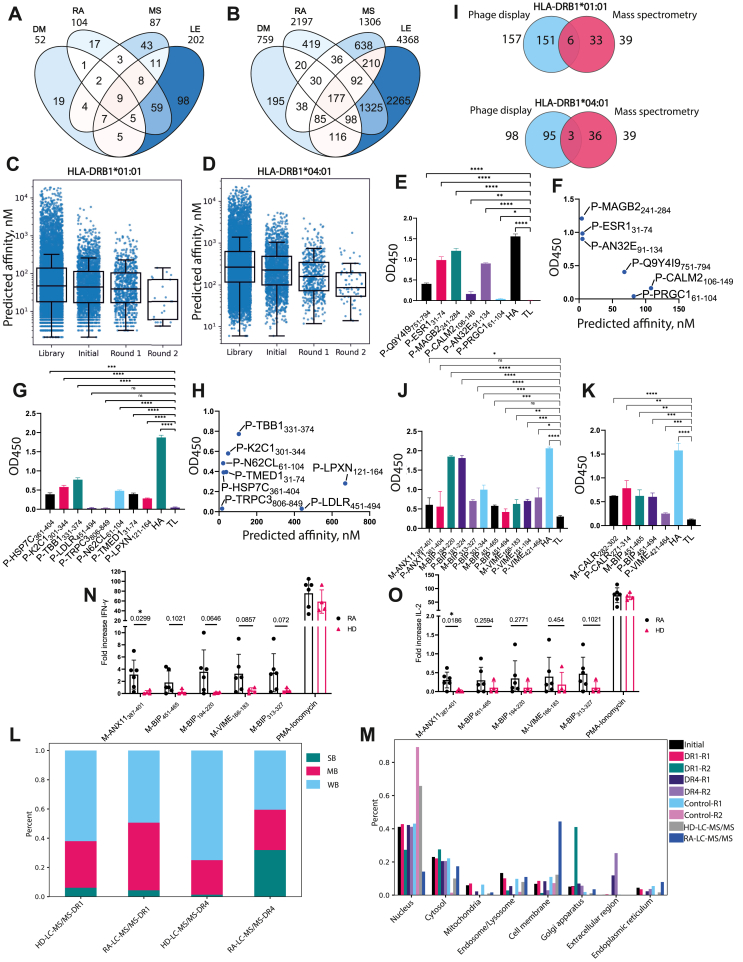
Identification of rheumatoid arthritis (RA)-associated autoantigenic peptides presented on HLA-DRB1∗01:01 and HLA-DRB1∗04:01. **(A)** The distribution of autoantigenic proteins associated with diabetes mellitus (DM), RA, multiple sclerosis (MS), and lupus erythematosus (LE) in designed library. **(B)** The distribution of autoantigenic peptides associated with DM, RA, MS, and LE in the designed library. **(C, D)** The predicted affinity of ligands significantly decreases along selection from the theoretical library to the second round of enrichment for (C) HLA-DRB1∗01:01 or (D) HLA-DRB1∗04:01. *X*-axis labels: Library, all ligands from the theoretical library; Initial, ligands successfully expressed in bacteriophages; Round 1, ligands passing the first selection round with HLA-DRB1∗01:01/HLA-DRB1∗04:01; Round 2, ligands passing the second selection round with HLA-DRB1∗01:01/HLA-DRB1∗04:01. Ligands selected during the negative control experiment are excluded from each group. Mann–Whitney's U-test *p*-values <0.002. **(E, G)** Recombinant HLA-DRB1∗01:01 (E) or HLA-DRB1∗04:01 (G) (150 nM) were incubated overnight with biotinylated recombinant trx-peptides (750 nM). **(F, H)** Experimentally obtained OD450 values of overnight binding to HLA-DRB1∗01:01 (F) or HLA-DRB1∗04:01 (H) were correlated with predicted affinities. HA, positive control (hemagglutinin HA_306-318_ fragment of influenza A virus in fusion with trx); TL, negative control (thioredoxin with S-G linker). The amount of bound biotinylated trx-peptide was determined with the addition of horseradish peroxidase (HRP)-streptavidin. Standard deviation is indicated. *p*-values are generated from a two-tailed student's *t*-test: ^∗∗∗∗^*p* < 0.0001, ^∗∗∗^*p* < 0.001, ^∗∗^*p* < 0.01, ^∗^*p* < 0.05; ^ns^, *p* > 0.05. **(I)** Venn diagrams of the distribution of peptides identified with phage display and liquid chromatography with tandem mass spectrometry (LC-MS/MS) for HLA-DRB1∗01:01 and HLA-DRB1∗04:01. **(J, K)** Recombinant HLA-DRB1∗01:01 (J) or HLA-DRB1∗04:01 (K) (150 nM) were incubated overnight with biotinylated trx-peptides (750 nM). Standard deviation is indicated. *p*-values are generated from a two-tailed student's *t*-test: ^∗∗∗∗^*p* < 0.0001, ^∗∗∗^*p* < 0.001, ^∗∗^*p* < 0.01, ^∗^*p* < 0.05; ^ns^, *p* > 0.05. **(L)** Distribution of LC-MS/MS identified peptides by affinity among RA patients and healthy donors (HDs). The cutoff for strong binders (SB) was below 50 nM, for moderate binders (MD) was 50–500 nМ, and for weak binders (WB) was above 500 nM. **(M)** Distribution of phage- and LC-MS/MS-selected peptides by cellular localization. The calculations were carried out for the results of selection for HLA-DRB1∗01:01 and HLA-DRB1∗04:01, as well as for two control rounds of selection without subtracting them from the results of selection for HLA-II. The cellular localization of the obtained antigens was determined using https://www.proteinatlas.org (subcellular localization section). Identified synthetic peptides were used for stimulation of CD4^+^ T cells from peripheral blood mononuclear cells of HLA-DRB1∗01:01-positive RA patients (*n* = 6) and healthy donors (*n* = 4) and detection of **(N)** IFN-γ or **(O)** IL-2 production. *p* values are generated from a two-tailed Welch's *t*-test. Autoantigenic peptides are depicted as “M/P-X”, where “M”, “P”, and “X” denote mass spectrometry, phage display, and UniProt name and residue numbers, respectively.

The efficiency of HLA-II ligand selection was assessed computationally by predicting peptide affinities for HLA-DRB1∗01:01 and HLA-DRB1∗04:01 using NetMHCIIpan v4.0 (https://services.healthtech.dtu.dk/services/NetMHCIIpan-4.0/). The comparison between virtually calculated affinities and the enrichment efficiency of peptides during successive stages of phage display revealed that clones with higher affinity for the corresponding HLA-II molecules were selected with the greatest efficiency (Fig. 1C and D). Screening of the library for binding of HLA-DRB1∗01:01 and HLA-DRB1∗04:01 identified 157 and 98 fragments of autoantigenic proteins, associated with RA according to AAgAtlas (Table S2). Among them, several specific peptides were previously shown to be presented by RA-associated HLA-II, for example, enolase_326–340_ (KRIAKAVNEKSCNCL), endoplasmic reticulum chaperone BiP_456–475_ (DNQPTVTIKVYEGERPLTKD),^1^ and collagen type I alpha chain_226-242_ (SRLPIIDVAPLDVGAPDT).^2^

The binding of individual peptides fused to bacterial thioredoxin (trx-peptides)^3^ to HLA-II molecules was assessed *in vitro* by ELISA. In particular, six antigenic peptides for HLA-DRB1∗01:01 and eight peptides for HLA-DRB1∗04:01 were selected for *in vitro* testing based on the results of phage display (Table S3). Most trx-peptides demonstrated statistically significant binding to HLA-DRB1∗01:01 (Fig. 1E) or HLA-DRB1∗04:01 (Fig. 1G) compared with negative control. The binding of trx-peptides to HLA-II correlated well with the predicted affinity (Fig. 1F,H).

Peptides were also tested for cell surface presentation using DC2.4 dendritic cells expressing HLA-DRB1∗01:01 or HLA-DRB∗04:01 (Fig. S1). They were incubated with a mix of corresponding trx-peptides, followed by cell lysis and immunoprecipitation of peptide–MHC complexes. As a result, fragments of calmodulin-2 CALM2_142-149_ (FVQMMTAK) from DC-HLA-DRB1∗01:01 and short transient receptor potential channel 3 TRPC3_835-849_ (SHSFNSILNQPTRYQ) from DC-HLA-DRB1∗04:01 were detected by LC-MS/MS and could be potentially new autoantigens associated with RA. Both are linked to calcium signaling/metabolism, with TRPC3 possibly involved in joint inflammation and protein citrullination.

To explore the HLA-II autoantigen ligands of HLA-DRB1∗01:01 and 04:01-positive RA patients, we conducted LC-MS/MS analysis of the immunopeptidome from monocyte-derived dendritic cells obtained from peripheral blood mononuclear cells. Most detected peptides both from patients and healthy donors were 14–16 aa long, typical for HLA-II peptides (Fig. S2). Overlapping peptides identified by LC-MS/MS were collated, resulting in a total of 39 peptides identified out of 846, with known humoral RA associations according to AAgAtlas (Table S4). Among them, 3.8% of peptides (6 of 157) overlapped with phage-selected peptides for HLA-DRB1∗01:01 and 3.1% (3 of 98) for HLA-DRB1∗04:01, respectively (Fig. 1I). RA-associated dual-selected autoantigenic peptides identified both by phage display and LC-MS/MS for RA patients are presented in Table S3. Additionally, several dual-selected peptides were associated with RA according to sources other than AAgAtlas, for example, annexin A2_37-52_ (RDALNIETAIKTKGVD). Besides, the LC-MS/MS analysis identified several peptides not selected by phage display, but overlapped with previously validated RA-relevant HLA-II epitopes, for example, with vimentin_66–78_ (SAVRLRSSVPGVR),^4^ histone H2B_63-74_ (MNSFVNDIFERI), and histone H4_28-40_ (DNIQGITKPAIRR),^5^ which elicited CD4^+^ T cell response in RA patients.

The binding of dual-selected peptides was further verified *in vitro.* Short peptides were extended to 15 aa, while longer peptides were used at their original lengths to be produced in recombinant form as trx-peptides (Table S3). Notably, P-VIME_421-464_ was identified in LC-MS/MS as a 44 aa fragment, similar to its phage display counterpart. Generally, all peptides were characterized as reliably binding HLA-DRB1∗01:01 (Fig. 1J) and HLA-DRB1∗04:01 (Fig. 1K).

Theoretical affinity calculations for an array of peptides obtained by LC-MS/MS showed that, in general, the HLA-II immunopeptidome is characterized by the presence of peptides with higher affinity to disease-associated HLA-II in patients with RA than in healthy donors (Fig. 1L). Among autoantigens selected by phage display and LC-MS/MS in RA patients, enrichment of the Golgi apparatus protein fraction was observed. Also, enrichment in peptides associated with membrane, endoplasmic reticulum, and extracellular protein fractions was revealed. Importantly, these compartments are involved in the endocytic antigen pathway used for exposure of HLA-II on the cell surface (Fig. 1M).

To evaluate the immunogenicity of identified peptides, we assessed IFN-γ and IL-2 production by incubating peripheral blood mononuclear cells of HLA-DRB1∗01:01-positive RA patients (*n* = 6) and healthy donors (*n* = 4), and of HLA-DRB1∗04:01-positive RA patients (*n* = 5) and healthy donors (*n* = 4) with dual-selected synthetic peptides (Table S3 and Fig. S3, 4). Among them, the fragment of annexin A11_387-401_ (SRAHLVAVFNEYQRM) induced a statistically significant difference in IFN-γ (*p* = 0.0299) and IL-2 (*p* = 0.0186) production between HLA-DRB1∗01:01-positive RA patients and healthy donors (Fig. 1N, O). The T cell-mediated association of this peptide with RA was reported in our study for the first time, although annexin A11 has previously been mentioned as a target for autoantibodies in autoimmune diseases.

In summary, we identified RA-associated autoantigenic peptides presented on HLA-DRB1∗01:01 and HLA-DRB1∗04:01 molecules using a dual synergistic approach. These peptides may be involved in RA pathogenesis and serve as potential targets for antigen-specific therapies. Our strategy offers a comprehensive tool for identifying autoimmunity-linked HLA-II peptide ligands.

## CRediT authorship contribution statement

**Irina A. Ishina:** Writing – review & editing, Writing – original draft, Visualization, Supervision, Software, Methodology, Investigation, Formal analysis, Data curation, Conceptualization. **Anton P. Zhiyanov:** Software, Methodology, Formal analysis, Data curation. **Inna N. Kurbatskaia:** Writing – review & editing, Writing – original draft, Methodology, Investigation, Conceptualization. **Azad E. Mamedov:** Methodology, Investigation, Conceptualization. **Stepan A. Nersisyan:** Software, Methodology, Formal analysis, Data curation. **Rustam H. Ziganshin:** Investigation. **Igor E. Eliseev:** Software, Formal analysis. **Yunna S. Petrusenko:** Software, Formal analysis. **Anastasia V. Nikonova:** Investigation. **Elizaveta S. Zhbanova:** Investigation. **Maria A. Salnikova:** Investigation. **Leyla A. Ovchinnikova:** Investigation. **Ilgar Z. Mamedov:** Investigation. **Alexey N. Davydov:** Software, Formal analysis. **Kamila S. Nurbaeva:** Investigation. **Tatiana A. Lisitsyna:** Writing – review & editing, Supervision. **Tatiana M. Reshetnyak:** Writing – review & editing, Supervision. **Alexander M. Lila:** Writing – review & editing, Supervision. **Evgeniy L. Nasonov:** Writing – review & editing, Supervision. **Yakov A. Lomakin:** Writing – review & editing, Methodology. **Alexey A. Belogurov:** Writing – review & editing, Methodology. **Hongkai Zhang:** Writing – review & editing, Methodology. **Alexander G. Tonevitskiy:** Writing – review & editing, Supervision. **Yury P. Rubtsov:** Writing – review & editing, Writing – original draft, Supervision, Resources, Conceptualization. **Alexander G. Gabibov:** Writing – review & editing, Supervision, Resources, Project administration, Funding acquisition, Conceptualization. **Maria Y. Zakharova:** Writing – review & editing, Writing – original draft, Supervision, Resources, Project administration, Methodology, Investigation, Conceptualization.

## Ethics declaration

The studies involving humans were approved by the Ethics Committee of the V.A. Nasonova Research Institute of Rheumatology (Protocol No. 3, dated February 2, 2023). The studies were conducted following the local legislation and institutional requirements. The participants provided their written informed consent to participate in this study.

## Funding

This article was prepared as a part of the fundamental scientific theme No. 122040400024-7 of the V.A. Nasonova Research Institute of Rheumatology. We would like to appreciate Bioresource Collection–Collection of SPF-Laboratory Rodents for Fundamental, Biomedical and Pharmacological Studies supported by the Ministry of Science and Higher Education of the Russian Federation (Contract No. 075-15-2021-1067). The research was performed in part within the framework of the “Creation of Experimental Laboratories of the Natural Sciences program” and the Basic Research program at HSE University. And this study was supported by the Russian Science Foundation (No. 22-14-00219).

## Conflict of interests

The authors declare that the research was conducted in the absence of any commercial or financial relationships that could be construed as a potential conflict of interest.
