# Supplementary material for: Autoantigenic peptide landscape of rheumatoid arthritis-associated HLA class II
Source: Genes Dis. 2024 Nov 26;12(4):101469. doi: 10.1016/j.gendis.2024.101469 (PMC11999199; doi:10.1016/j.gendis.2024.101469)
Supplement: Multimedia component 1 [file mmc1.docx]

Autoantigenic peptide landscape of rheumatoid arthritis-associated HLA class II


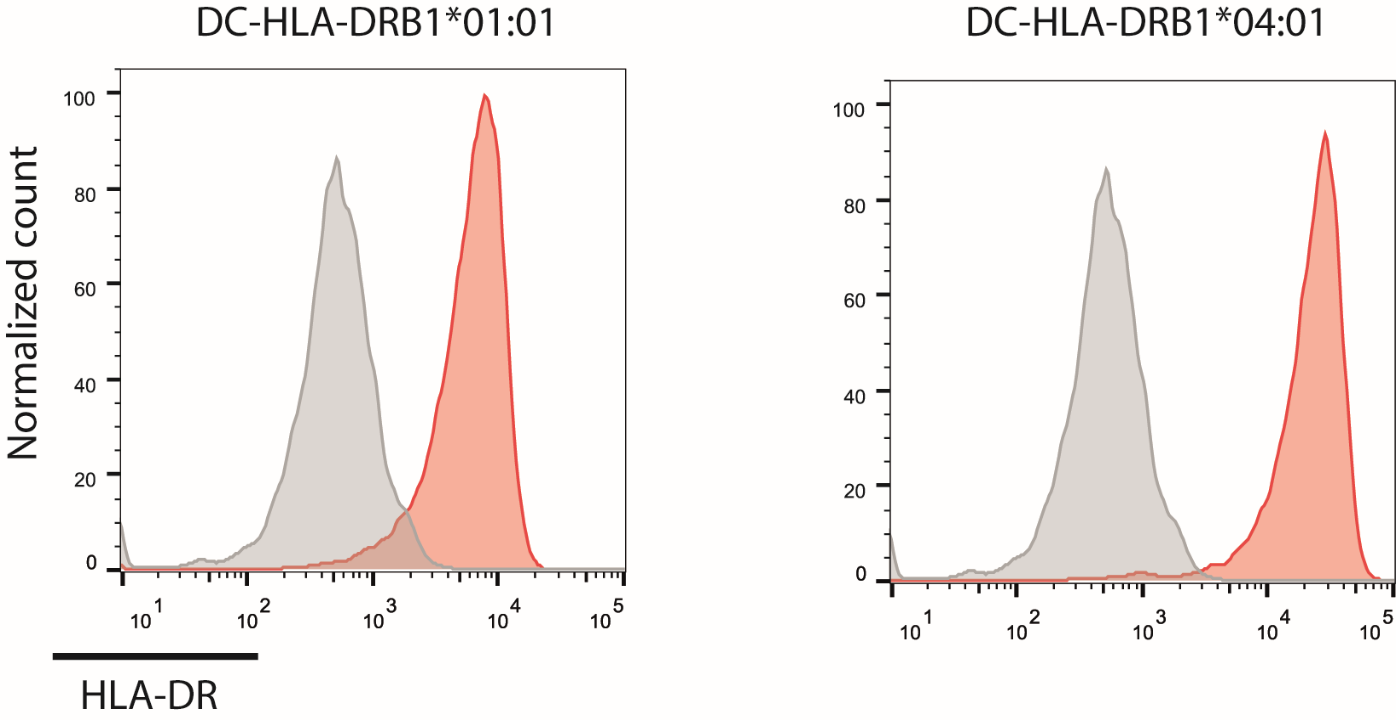


**Supplementary figure 1. DC-DR cell lines expressing HLA-DRB1*01:01 or HLA-DRB1*04:01 molecules.** DC2.4 cell line was transduced with lentiviral particles coding HLA-DRB1*01:01 or HLA-DRB1*04:01. Grey histogram represents non-transduced DC2.4 cell line stained with anti-HLA-DR. Surface staining for the expression of HLA-DR molecules was carried out with flow cytometry.


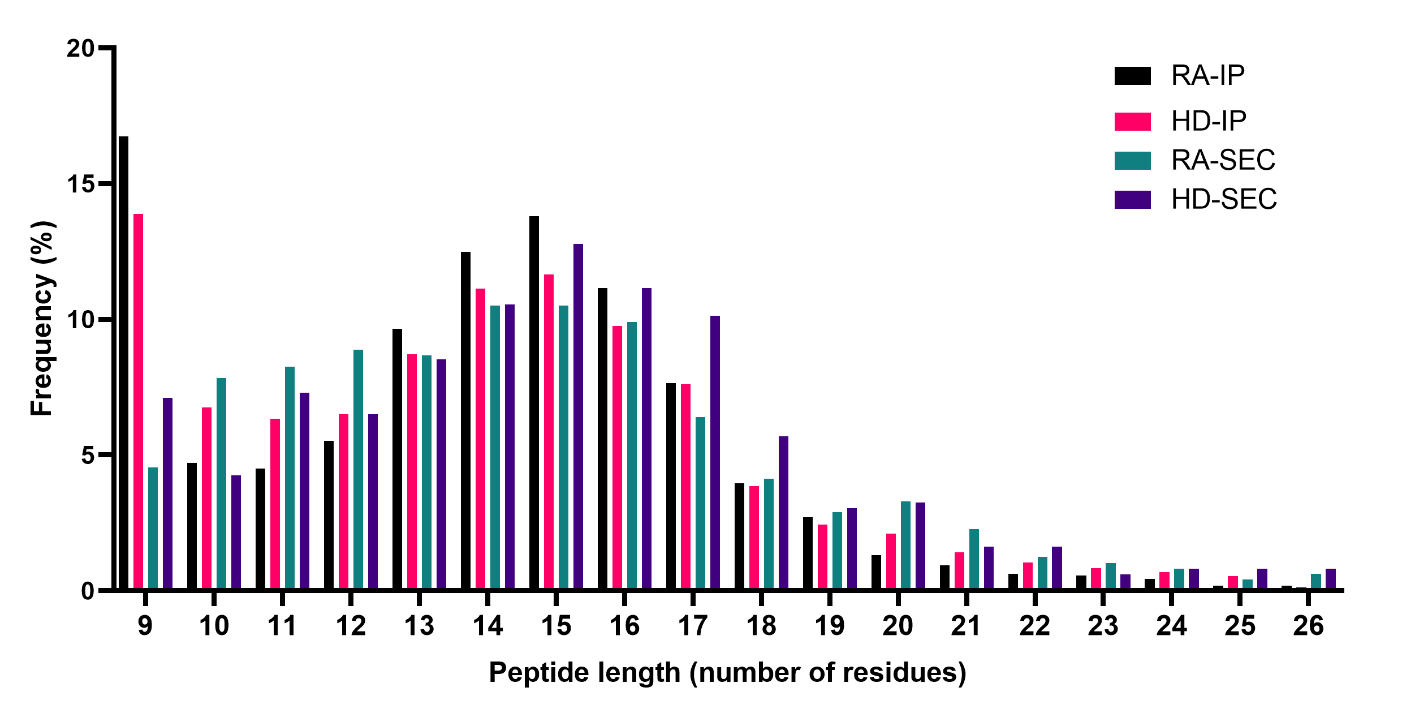


**Supplementary figure 2. Length distribution of peptides eluted from HLA-II molecules.** Samples were acquired with IP (immunoprecipitation) – RA-IP, HD-IP or SEC (size-exclusion chromatography) – RA-SEC, HD-SEC.


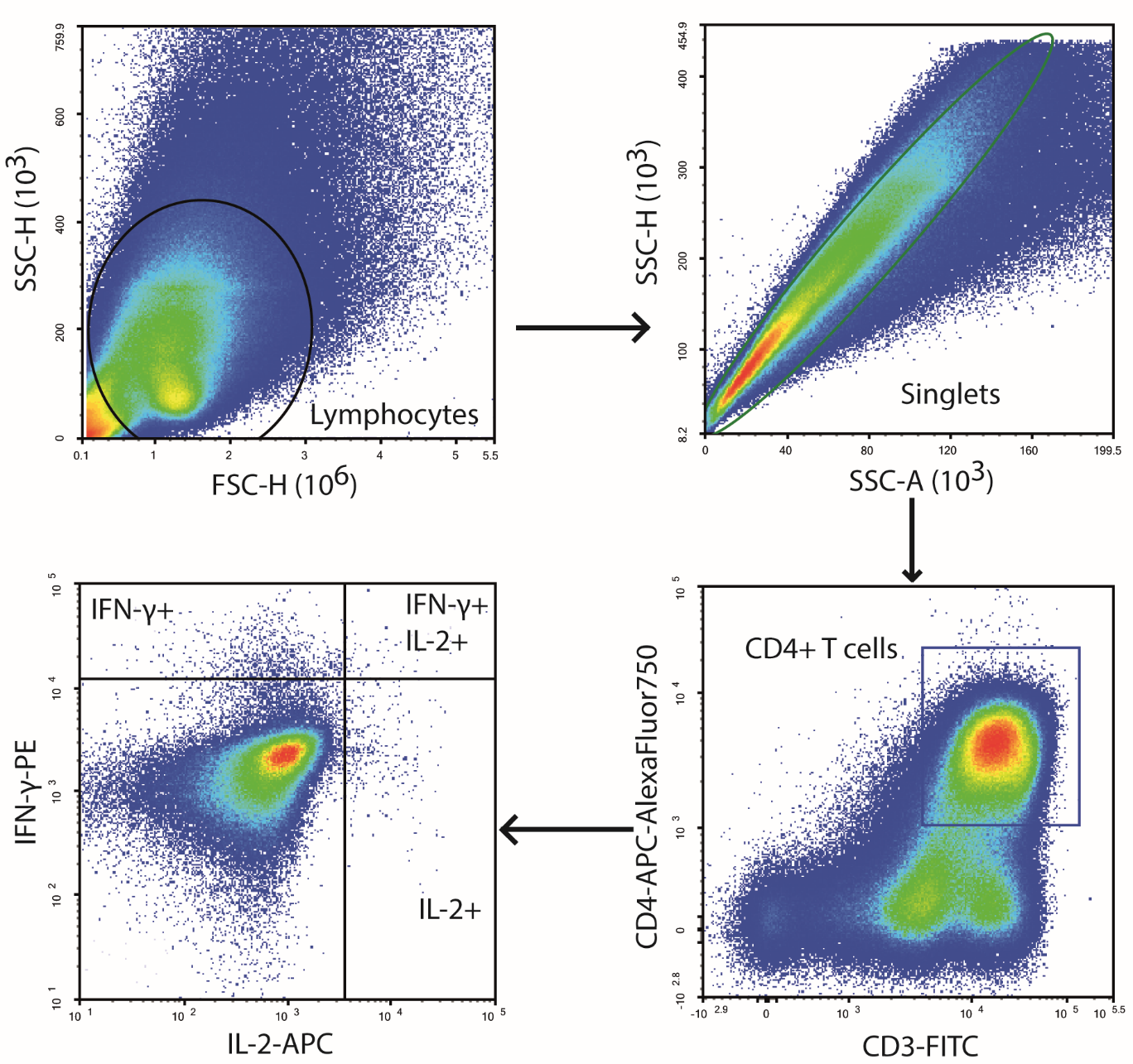


**Supplementary figure 3. Gating strategy for assessing the production of IFN-γ and IL-2 by CD4^+^ T cells.** PBMC were sequentially gated on lymphocytes, singlets, CD4^+^ T cells. The expression of IFN-γ and IL-2 was analyzed in CD4^+^ T cells.


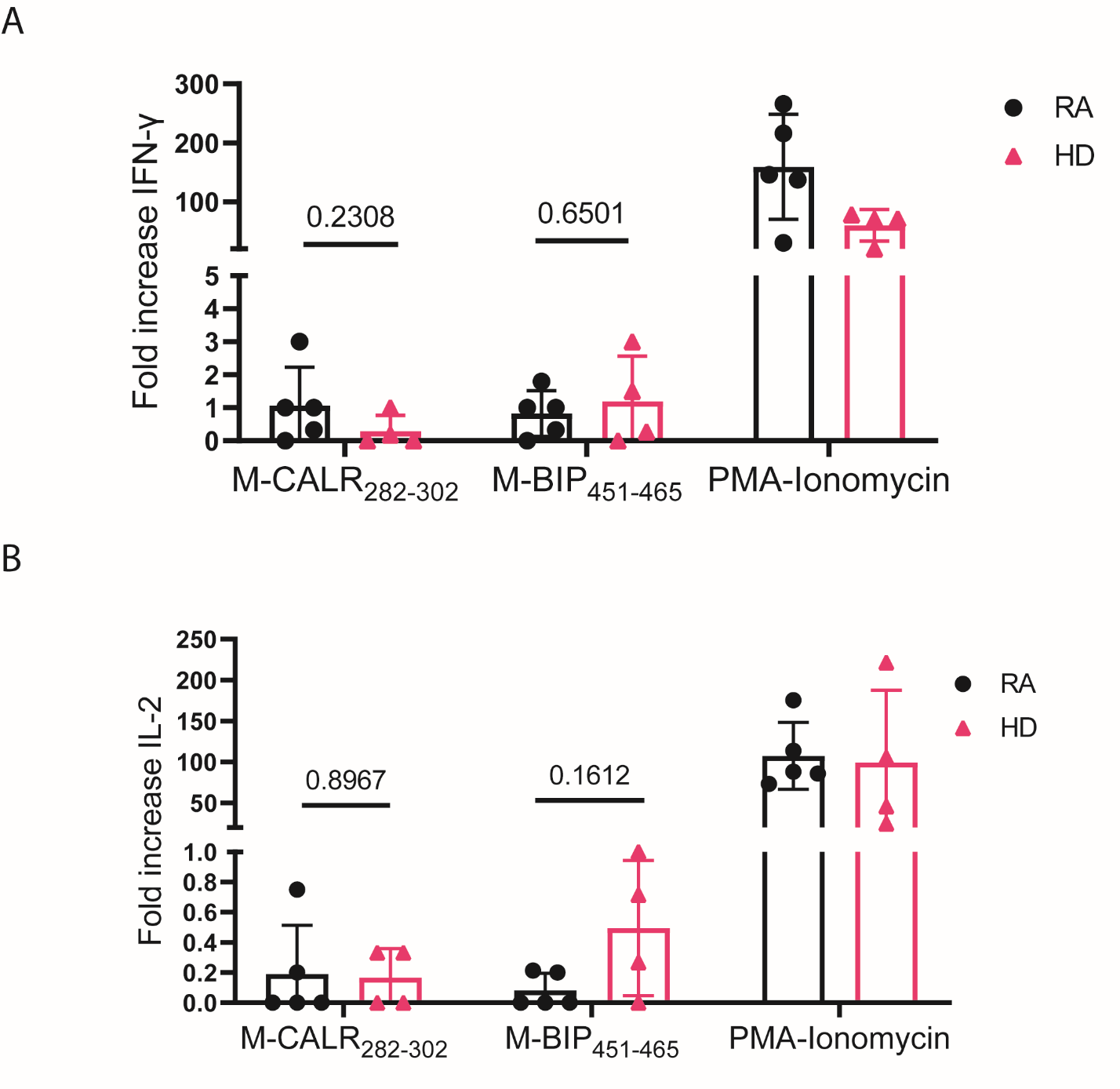


**Supplementary figure 4. CD4^+^ T cell response for peptides selected with phage display and mass spectrometry for HLA-DRB1*04:01 molecule.** (A) Synthetic peptides were used for stimulation of CD4^+^ T cell from PBMC of HLA-DRB1*04:01-positive RA patients (n=5) and HD donors (n=4) and detection of IFN-γ or (B) IL-2 production. P values are generated from a two-tailed Welch’s t test.


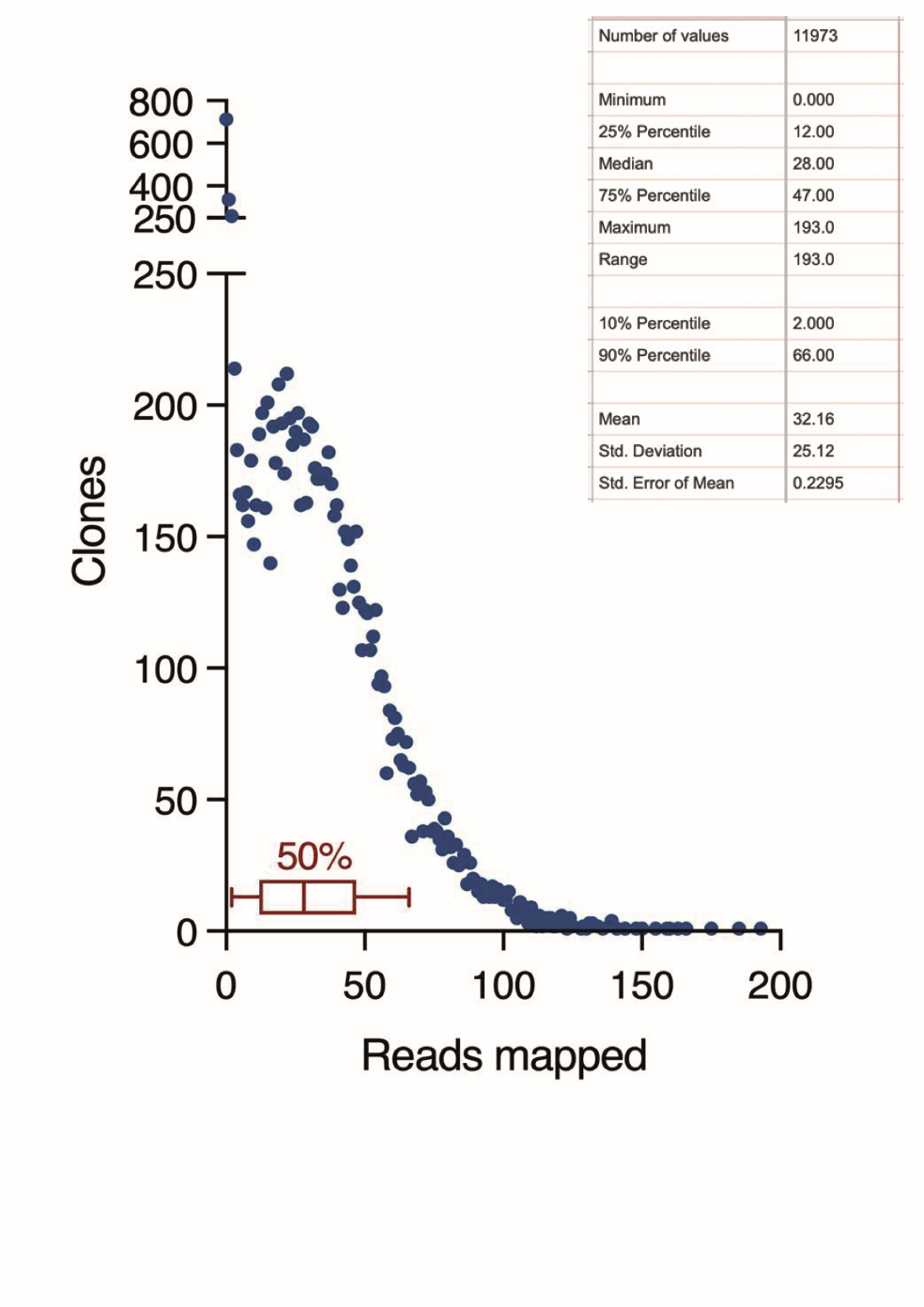


**Supplementary figure 5. Quality analysis of the obtained synthetic DNA library observed by Illumina sequencing.** 94% of all clones (11 261 of 11 973) were detected. Whiskers on the box plot show 10th and 90th percentiles.


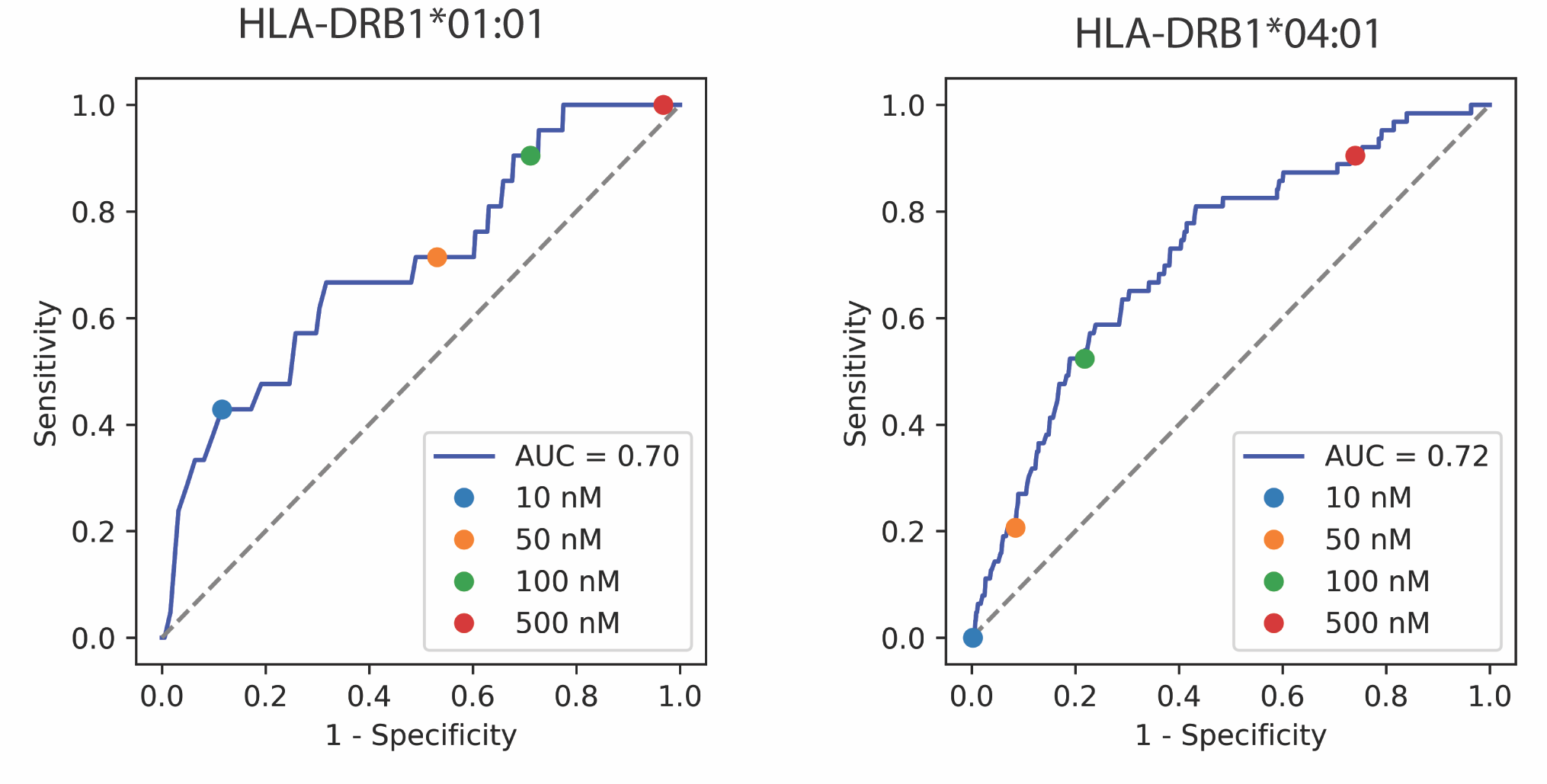


**Supplementary figure 6. ROC curves for ligands, not passing any selection vs passing the second selection.** Colored points additionally mark frequently used thresholds (10, 50, 100, 500 nM). At a given affinity threshold, sensitivity is defined as the fraction of predicted tight binders within peptides passing both selection rounds and specificity is defined as the fraction of predicted weak binders not passing the second selection round.


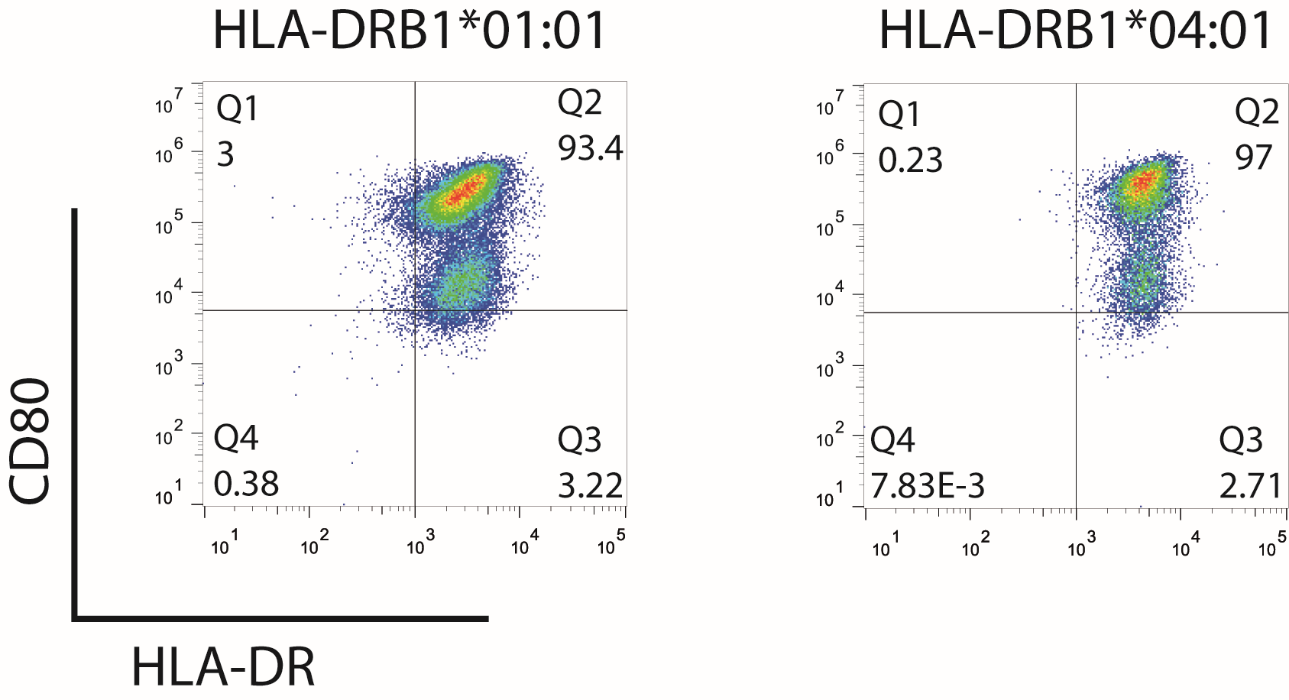


**Supplementary figure 7. HeLa cell lines expressing CD80 and HLA-DRB1*01:01 or HLA-DRB1*04:01 molecules.** HeLa cells were successively transduced with lentiviral particles coding for CD80 and HLA-DRB1*01:01 or HLA-DRB1*04:01. Surface staining for the expression of CD80 and HLA-DR molecules on HeLa cell lines was carried out with flow cytometry.
