# Supplementary material for: Autoantigenic peptide landscape of rheumatoid arthritis-associated HLA class II
Source: Genes Dis. 2024 Nov 26;12(4):101469. doi: 10.1016/j.gendis.2024.101469 (PMC11999199; doi:10.1016/j.gendis.2024.101469)
Supplement: Multimedia component 2 [file mmc2.docx]

**Materials and Methods**

Individuals

The study included 15 patients with a reliable diagnosis of RA and with specific HLA-II alleles (HLA-DRB1*01:01 or HLA-DRB1*04:01), of whom 14 were women and 1 was man. The average age of the patients was 50±12.61 years. 11 healthy donors (HDs) with specific HLA-II alleles (HLA-DRB1*01:01 or HLA-DRB1*04:01) without rheumatic disorders were included in the control group, of whom 7 were women and 4 were men. The average age of the donors was 36±9.14 years (Supplementary table 5). The 2010 ACR/EULAR criteria were used to diagnose RA^21^. All patients signed an informed consent to participate in the study. The study was approved by the local ethics committee at the V.A. Nasonova Research Institute of Rheumatology (Protocol No. 3, dated February 2, 2023). All patients underwent a conventional examination, and immunological studies included C-reactive protein (CRP), rheumatoid factor (RF), and antibodies to citrullinated cyclic peptide (ACCP). The serum concentration of CRP (the upper limit of the norm is 5 mg/L) and RF IgM (the upper limit of the norm is 15 IU/mL) was determined by immunonephelometry on a BN ProSpec analyzer (Siemens). Quantitative determination of ACCP in blood serum was carried out using the electrochemiluminescent method on the Cobas e411 analyzer (Roche, the upper limit of the norm is 17 Units/mL) and the enzyme-linked immunosorbent assay (ELISA) method of Axis Shield Diagnostics (the upper limit of the norm is 5 Units/mL).

Autoantigen phage library construction, expression, and purification

Each autoantigenic sequence was computationally divided into 132 nt (nucleotides) coding sequences, optimized for prokaryotic expression. To enable amplification, 17 nt and 20 nt adapters were added to 5’ end 3’ end of each sequence, respectively. For uniform distribution of peptide sequences and efficient DNA amplification emulsion polymerase chain reaction (PCR) was used to obtain final autoantigen library. Amplified library was cloned into fd bacteriophage vector fADL-1e in fuse with bacteriophage coat protein p3 and transformed into E. coli TG-1 cells. The resulting peptide phage library was presented as 44 amino acid (aa) peptides with 14 aa overlap. The obtained colonies were eluted, resuspended in cryopreservation medium (2xYT, 10% glycerol) and stored at −80°C until further usage. Phage virions were obtained using the established protocol^24^. The bacterial stock was inoculated into growth medium (2xYT, 50 µg/ml of Kanamycin) and incubated at 37°C overnight. The overnight culture was diluted 1:100 and incubated at 30°C for 24 hours. Recombinant bacteriophages were purified by double Polyethylene glycol (PEG 6000) precipitation^25^. The concentration of bacteriophage particles was determined with ELISA. Purified phage particles were captured on the immunosorbent plate (Nunc) with immobilized anti-M13 antibodies (Sigma-Aldrich) and quantitated with HRP-conjugated anti-M13 (GE Healthcare), anti-FLAG antibodies (Sigma-Aldrich). ELISA was performed using standard protocol. Sequencing data indicated that 94% of the theoretically chosen sequences were present in resulting synthetic DNA library (Supplementary figure 5).

Expression of recombinant HLA-II protein

Recombinant HLA-II proteins HLA-DRB1*01:01 and HLA-DRB1*04:01 with empty peptide-binding groove used for phage display selection were produced in Drosophila melanogaster S2 cells as previously described^26^. Briefly, the cell lines were created by stable transfection of HLA-DR (HLA-DRB1*01:01 and HLA-DRB1*04:01) α and β chains in pMT-V5/His and pRmHa backbones with pCoBlast (Invitrogen) for subsequent selection with blasticidin. The expression of HLA-II was induced with 1 mM Cu^2+^ upon reaching density of 10 million cells/mL followed by 7 days culturing in SF900 III media (Gibco) at 27°C with shaking. The concentrated cell culture supernatant was purified with Ni-Nitrilotriacetic acid (Ni-NTA) (Qiagen) and MonoQ columns (GE Healthcare) with 0−1 M NaCl gradient. HLA-II with CLIP in peptide-binding groove and HLA-DM recombinant proteins were expressed in HEK293F cells in FreeStyle medium (Gibco) for 7 days following transient transfection of HLA α and β chains in pFUSE vector additionally encoding constant fragment of human immunoglobulin heavy chain (Fc) for complex heterodimerization and higher expression yields. Recombinant protein was purified with Protein G affinity column (GE Healthcare) by elution with 100 mM Gly-HCl, pH 2.5 and neutralization of eluate by 1 M Tris-HCl, pH 8.0. The obtained proteins were stored in Tris-buffered saline (TBS) buffer (50 mM Tris-Cl, 150 mM NaCl, pH 7.5) at 4°C. CLIP peptide connected to the N-terminus of the HLA-DR β chain was cut with 20 U/mg of biotinylated thrombin (Millipore) for 1 hour at 25°C just before use. Thrombin was removed by incubation with streptavidin-agarose for 15 min at 25°C, the flowthrough HLA-II protein was transferred to the reaction buffer using Amicone 10 kDa centrifuge unit (Millipore).

Selection of autoantigenic peptides binding HLA-II molecules by phage display

Recombinant HLA-II and HLA-DM proteins were incubated in equimolar quantity (50 pmol) with 2.5x10^9^ phages in phage binding buffer (50 mM sodium citrate, 150 mM NaCl, 2 mM ethylenediaminetetraacetic acid (EDTA), 0.2% NP-40, 1 mM phenylmethylsulfonyl fluoride (PMSF), pH 6.0) in 50 μL at 25°C for 48 hours. Following the incubation, the mixture was diluted to 1 mL with phage binding buffer containing 0.5% dry milk and applied to immunotube (Nunc) for 2 hours at 37°C coated with L243 antibody and blocked with 5% dry milk in advance. Multiple sequential washing steps with 10 portions of 4 mL of phosphate-buffered saline (PBS) with 0.1% Tween 20 and 10 portions of 4 mL of PBS were performed. The elution of the bounded phages was implemented by incubation with 1.5 mL of 100 mM Gly-HCl, pH 2.5 at 25°C for 10 min with shaking followed by neutralization with 2 M Tris-HCl, pH 8.0. The eluted phages were subjected to further infection of E. coli TG-1 cells in the log phase (OD_600_ = 0.6) during 30 min without shaking. The resulting peptide pools at each display step were characterized by NGS.

Virtual binding assessment of autoantigenic peptides

Each 44-mer peptide of the autoantigen library was split into all possible 15-mers peptides. Then, binding affinities between the resulting 15-mers and HLA-DRB1*01:01/HLA-DRB1*04:01 molecules were predicted using NetMHCIIpan v4.0 (https://services.healthtech.dtu.dk/services/NetMHCIIpan-4.0/). The binding affinity between a 44-mer peptide and an HLA-II molecule was then defined as a minimum affinity across all 15-mer peptides of the parental peptide. The previously developed T-CoV framework was used to implement the aforementioned procedure. ROC curves were constructed and visualized using scikit-learn and seaborn packages for Python programming language, respectively. Using a threshold binding affinity value (e.g., 50 nM), we defined sets of tightly binding peptides (IC50 lower than the threshold) and weakly binding peptides (IC50 higher than the threshold). This allowed to estimate the sensitivity (the fraction of tight binders within peptides following two selection rounds) and specificity (the fraction of weakly binding peptides washed out following the second selection round) of the phage display rounds for HLA-DRB1*01:01 or HLA-DRB1*04:01. The predicted binding affinity threshold was then plotted with the resulting sensitivity/specificity values as a receiver operating characteristic (ROC) curve (Supplementary figure 6). The areas under the ROC curves (ROC AUC) for HLA-DRB1*01:01 and HLA-DRB1*04:01 were 0.7 and 0.72, respectively, indicating a moderate and significant degree of concordance between the phage display experimental results and the computational estimation.

HLA typing

To determine the HLA-II genotype of the donor peripheral blood mononuclear cells (PBMC) were resuspended in Qiazol reagent (Qiagen), total RNA was extracted according to manufacturer’s protocol and used for next-generation sequencing (NGS) library preparation. First strand cDNA synthesis was performed with specific primers corresponding to a highly conserved regions of HLA-A, HLA-B, HLA-C, HLA-DQB and HLA-DRB genes. Next, cDNA was amplified with 8 primer mixes covering exons 2-4 of HLA-I and exons 2-3 of HLA-II in 8 separate PCR reactions for each individual. Eight products of the first PCR reactions for each donor were mixed and amplified with IDT for Illumina UD Indexes kit (Illumina, USA). Libraries were purified with Ampure XP beads (Beckman Coulter, USA) according to manufacturer's protocol. Sequencing was performed on Illumina MiSeq (Illumina, USA) paired end, 250. Bioinformatic data analysis was performed in R IDE using a custom algorithm (https://github.com/asya-minervina/HLA) that aligns raw sequencing reads to HLA sequence database (extracted from IMGT/HLA database), get rid of PCR and sequencing errors and assemble HLA genotype of individual.

NGS and data analysis

Two-round PCR was employed to prepare NGS libraries. Purified phage particles were amplified with forward primer (5’-GTCTCGTGGGCTCGGAGATGTGTATAAGAGACAGACTAACGAGGGCAA-3’) and reverse primer (5’-TCGTCGGCAGCGTCAGATGTGTATAAGAGACAGCAACAGTTTCGGCTT-3’) using the maximum number of amplification rounds before the appearance of PCR product assessed by agarose gel electrophoresis. The resulting product was cleaned using Agencourt AMPure XP magnetic beads (Beckman Coulter) according to manufacturer’s instructions and amplified with forward (5’-AATGATACGGCGACCACCGAGATCTACACXXXXXXXXTCGTCGGCAGCGTCAG-3’) and reverse primers (5’-CAAGCAGAAGACGGCATACGAGATXXXXXXXXGTCTCGTGGGCTCGG-3’) carrying specific sequences of indexes. The obtained libraries were cleaned with magnetic beads and sequencing was performed on Illumina MiSeq (Illumina, USA). MiXCR software (www.mixcr.com) was utilized to analyze the raw data. Since the data does not consist of immune receptor sequences and lacks the CDR3 region, custom references were constructed to simulate the crucial anchor points (the start and end of the "CDR3") required for the correct functionality of MiXCR. In this process, constant sequences surrounding the peptide were employed as V and J segments, while diverse peptide sequences were used as the CDR3.

Recombinant thioredoxin - peptide fused proteins (trx-peptides) production

The sequences of antigenic peptides (12 to 44 aa) were added to the C-terminus of bacterial trx through serine-glycine linker in pET32CH construct carrying His-tag for purification. The negative control (TL) encoded only linker and trx sequences in the construct. Trx-peptides were produced in E. coli BL21(DE3) cells in soluble form and purified sequentially with Ni-NTA (Qiagen) and Superdex 30 (GE Healhcare) columns. The purity of the proteins was analyzed by Laemmlie PAGE and was assessed as more than 90%. Trx-peptides were chemically biotinylated with EZ-Link Sulfo-NHS-LC-biotin (Thermo Fisher Scientific) in molar ratio 1:20 for 1 hour at 25°C and then purified from unbound label using Amicone 10 kDa centrifuge unit (Millipore). The labeling was confirmed by WB with Streptavidin-HRP staining. Proteins were stored at −20°C in PBS.

Determination of trx-peptide/HLA-II binding

To assess peptide binding to HLA-II molecule biotinylated trx-peptides (750 nM) were incubated with HLA-II recombinant protein carrying Fc fragment (HLA-DRB1*01:01 or HLA-DRB1*04:01) (150 nM) at 37°C in PBS in 50 μL overnight. HLA-II/trx-peptide complexes were captured on the immunosorbent plate (Nunc) with immobilized L243 antibody. HLA-II bound biotinylated peptides were quantitated with HRP-conjugated streptavidin. HA peptide (hemagglutinin fragment of influenza A virus -HA_306-318_ in fusion with trx) was used as positive control. TL peptide (trx with S-G linker without fused peptide) was used as a negative control.

Identification of autoantigenic peptides presented on HLA-II molecules of human Monocyte-derived Dendritic cells (Mo-DCs) with LC-MS/MS

PBMC were extracted from whole blood using Ficoll gradient centrifugation, resuspended in RPMI advanced medium with 10% fetal bovine serum (FBS), 100 U/mL penicillin, 100 μg/mL streptomycin, 0.25 μg/mL Amphotericin B, and 2 mM GlutaMAX (Gibco) and plated to 25 cm^2^ cultural flasks in 6x10^6^ cell/mL concentration. Following 2 hours incubation unbound cells were removed and media was changed to RPMI full containing IL-4 (100 ng/mL) and granulocyte-macrophage colony-stimulating factor (GM-CSF) (50 ng/mL) (StemCell Technologies). Mo-DCs were differentiated for 6 days with change of media every 2 days. Bacterial lipopolysaccharide (LPS) (10 μg/mL) was added after 6 days and Mo-DCs were cultivated for 24 hours for maturation. Next, Mo-DC or DC2.4 (loaded with trx-peptides) were unbound by cell scrapper, lysed in PBS with 0.25% of sodium deoxycholate in presence of complete EDTA-free inhibitors (Roche), PMSF, Pepstatin, EDTA for 1 hour at 4°C following centrifugation at 16.000g for 20 min. Cleared cell lysates were incubated with Protein A Sepharose column for 30 min at 4°C. The flow-through was incubated overnight at 4°C with Protein A Sepharose resin covalently bound to L243 antibody. The resin was prepared according to the published protocol. The resin was washed with 40 volumes of PBS. pMHC (peptide–MHC) complexes were eluted with 0.1% Trifluoroacetic acid (TFA) and heated at 70°C for 10 min for elution of peptides. Alternatively, cell lysates were applied to Superdex75 (GE Healthcare) column. High-molecular fractions were collected, the presence of HLA-DR molecules was verified by ELISA with immobilized L243 antibody and detected with rabbit anti-HLA-II polyclonal serum and HRP-conjugated anti-rabbit antibody (Sigma). The fractions were lyophilized and desalted using SDB-RPS StageTips as it was described earlier^30^. LC-MS/MS analysis was conducted using the Q Exactive HF benchtop Orbitrap mass spectrometer coupled to the Ultimate 3000 Nano LC System via a nanoelectrospray source (Thermo Fisher Scientific). The HPLC system was configured in a trap-elute mode. Peptides were loaded on an Acclaim PepMap 100 (100 μm × 2 cm) trap column and separated on an Acclaim PepMap 100 (75 μm × 50 cm) column (Thermo Fisher Scientific). Correlation of MS/MS spectra with peptide sequences was made using PEAKS Studio 8.0 software. The false discovery rate (FDR) for peptide-spectrum matches was set to 0.01 and was determined by searching a reverse database. Peptide identification was performed with an allowed initial precursor mass deviation up to 10 ppm and an allowed fragment mass deviation of 0.05 Da.

Transduction of DC2.4 and HeLa cell lines

Lentiviral particles were produced by PEI co-transfection of HEK293T cells with plasmids encoding gene of interest and packaging plasmids. The medium was changed after 6 hours post-transfection to Opti-MEM supplemented with GlutaMAX, Sodium Pyruvate and 5% FBS (Gibco). Supernatant was collected 48 hours post-transfection. Medium containing lentiviral particles and polybrene (Merck) at 10 μg/mL were added to cell line in 6 well plate and centrifuged at 1,200g for 90 min at 30°C. The following day culture medium was changed to usual medium. The expression of CD80 and HLA-DR was assessed with NovoCyte flow cytometer (ACEA) using fluorophore-conjugated antibodies: anti-human CD80-PE (clone W17149D, Biolegend) and anti-human HLA-DR-APC (clone L243, Biolegend).

T cell *in vitro* expansion and intracellular IFN-γ, IL-2 staining

The methodology was adapted from the published protocol. PBMC were extracted from whole blood using Ficoll gradient centrifugation, counted and resuspended at 0.5 million cells/mL in RPMI full supplemented with IL-4 (100 ng/mL), GM-CSF (50 ng/mL), and Fms-related tyrosine kinase 3 ligand (Flt3-L) (50 ng/mL) (StemCell Technologies). 200 μL of cell suspension were added to 96-well round bottom cell plate (Corning) and incubated for 24 hours. Next, half of the media was changed to final concentration of 1 μM of each of pooled synthetic peptides and 10 μg/mL of LPS and incubated for 24 hours. Next, half of the media was changed to final concentration of IL-2 (10 IU/mL), IL-7 (10 ng/mL), and IL-15 (10 ng/mL). Then media was changed every 2–3 days. Last media change excluded growth factors. On day 10, PBMC were collected and resuspended at 1 million cells per well in AIM-V medium (Gibco) supplemented with AlbuMAX (Gibco) with final concentration of 1 μM of tested synthetic peptide and HeLa expressing CD80 and HLA-II (Supplementary figure 7) in 96-well round bottom plate. After 3 hours of incubation Brefeldin A (Biolegend) was added at concentration 10 μg/mL. For positive control PBMC were stimulated with 50 ng/mL PMA (Merck) and 1 μg/mL Ionomycin (Merck) for 2 hours, then Brefeldin A (Biolegend) was added at concentration 10 μg/mL. PBMC were incubated for 14 hours after Brefeldin A addition before intracellular cytokine staining. PBMC were washed with PBS and blocked with 5% normal mouse serum (NMS) (Thermo Fisher Scientific). Cells were stained with anti-human CD3-FITC (clone OKT3, Biolegend) and CD4-APC-AlexaFluor750 (clone S3.5, Thermo Fisher Scientific) antibodies for 15 min at 4°C. Then cells were washed with PBS and fixed with 2% paraformaldehyde in PBS for 20 min at 4°C. Cells were washed, blocked with 5% NMS, and stained with anti-human IFN-γ-PE (clone 4S.B3, Biolegend) and anti-human IL-2-APC (clone MQ1-17H12, Biolegend) antibodies for 40 min at 4°C in PBS supplemented with 0.2% saponin (Merck). Cells were analyzed with NovoCyte flow cytometer (ACEA).

Statistical analysis

Statistical analysis was performed with GraphPad Prism and Python. Statistical test employed is denoted in each figure legend; * indicates p < 0.05, ** p < 0.01, *** p < 0.001, **** p < 0.0001.
